# Improvement of leaf K^+^ retention is a shared mechanism behind CeO_2_ and Mn_3_O_4_ nanoparticles improved rapeseed salt tolerance

**DOI:** 10.1007/s44154-022-00065-y

**Published:** 2022-11-08

**Authors:** Yanhui Li, Jin Hu, Jie Qi, Fameng Zhao, Jiahao Liu, Linlin Chen, Lu Chen, Jiangjiang Gu, Honghong Wu, Zhaohu Li

**Affiliations:** 1 MOA Key Laboratory of Crop Ecophysiology and Farming System in the Middle Reaches of the Yangtze River, College of Plant Science & Technology, Huazhong Agricultural University, Wuhan, 430070 China; 2 Hubei Hongshan Laboratory, Wuhan, 430070 China; 3 College of Science, Huazhong Agricultural University, Wuhan, 430070 China; 4 College of Agronomy and Biotechnology, China Agricultural University, Beijing, 100083 China

**Keywords:** Nanomaterials, TEM imaging, Gene expression, Reactive oxygen species, K^+^/Na^+^ ratio

## Abstract

Salinity is a global issue limiting efficient agricultural production. Nanobiotechnology has been emerged as an effective approach to improve plant salt tolerance. However, little known is about the shared mechanisms between different nanomaterials-enabled plant salt tolerance. In this study, we found that both PNC [polyacrylic acid coated nanoceria (CeO_2_ nanoparticles)] and PMO (polyacrylic acid coated Mn_3_O_4_ nanoparticles) nanozymes improved rapeseed salt tolerance. PNC and PMO treated rapeseed plants showed significantly fresh weight, dry weight, higher chlorophyll content, Fv/Fm, and carbon assimilation rate than control plants under salt stress. Results from confocal imaging with reactive oxygen species (ROS) fluorescent dye and histochemical staining experiments showed that the ROS over-accumulation level in PNC and PMO treated rapeseed was significantly lower than control plants under salt stress. Confocal imaging results with K^+^ fluorescent dye showed that significantly higher cytosolic and vacuolar K^+^ signals were observed in PNC and PMO treated rapeseed than control plants under salt stress. This is further confirmed by leaf K^+^ content data. Furthermore, we found that PNC and PMO treated rapeseed showed significantly lower cytosolic Na^+^ signals than control plants under salt stress. While, compared with significantly higher vacuolar Na^+^ signals in PNC treated plants, PMO treated rapeseed showed significantly lower vacuolar Na^+^ signals than control plants under salt stress. These results are further supported by qPCR results of genes of Na^+^ and K^+^ transport. Overall, our results suggest that besides maintaining ROS homeostasis, improvement of leaf K^+^ retention could be a shared mechanism in nano-improved plant salt tolerance.

**Supplementary Information:**

The online version contains supplementary material available at 10.1007/s44154-022-00065-y.

## Introduction

Soil salinity issue is a limiting factor for efficient agricultural production. As an important cash crop, rapeseed is a source of vegetable oil. About 16% edible oil products are made from rapeseed (Ankit et al., 2021). It also plays important role in providing fodders to livestock (Mejicanos et al., 2016) and even can be used as vegetables (Ankit et al., 2021). However, soil salinity limits rapeseed yield and quality. This could be more severe in semi-arid area, especially the one with high salinity such as Xinjiang Province at China. Improving rapeseed salt tolerance could not only alleviate salinity resulted yield and quality penalty, but also accelerate its cultivation at semi-arid area with soil salinity issue, which could allow more arable land to be used to grow the cereal crops. Many efforts, from breeding (Ylvisaker, 1982), seed priming (Pace et al., 2012) to field management (Gao et al., 2022), have been tried to improve rapeseed salt tolerance. However, the progress does not meet people’s expectation. Introducing new techniques such as plant nanobiotechnology to improve rapeseed salt tolerance should be encouraged.

Plant nanobiotechnology approach is an emerging technique which can improve plant stress tolerance and thus can benefit efficient agricultural production (Wu and Li, 2022a; Zhao et al., 2020). Nanomaterials have been widely used to improve salt tolerance in many plant species, including maize (Liu et al., 2022; Oliveira et al., 2016), rice (Zhou et al., 2021), wheat (Saad-Allah and Ragab, 2020), barley (Karami and Sepehri, 2018), cotton (An et al., 2020; Liu et al., 2021), rapeseed (Khan et al., 2021; Li et al., 2022a, b; Rossi et al., 2016, 2017), cucumber (Chen et al., 2022; Lu et al., 2020), grapevine (Gohari et al., 2021) and Arabidopsis (Wu et al., 2018a). However, the possible commonly shared mechanisms in nano-improved plant salt tolerance are rarely discussed, especially among different nanomaterials. It is suggested that increase of ROS scavenging ability might be one of the commonly employed mechanisms underlying CeO_2_ nanoparticles improved plant salt tolerance (Khan et al., 2022). While besides ROS homeostasis, plant’s ability to maintain K^+^/Na^+^ ratio is a hallmark for its salt tolerance. Whether the mechanisms related to maintain K^+^/Na^+^ ratio homeostasis could be shared between salt stressed plants treated with different nanomaterials is still unknown.

CeO_2_ and Mn_3_O_4_ nanoparticles are known nanozymes having ROS scavenging ability (Wu and Li, 2022b). As above-mentioned, both of the nanoparticles showed good potential in improving plant salt tolerance (Chen et al., 2022; Li et al., 2022a, b; Lu et al., 2020; Wu et al., 2018a). However, as a rare earth element, cerium (Ce) is a heavy metal, which can rise public concerns about biosafety issues. Although technically, manganese (Mn) is also a heavy metal, but it is an essential micronutrient for plants. Mn-fertilizers are also used in agriculture (Deng et al., 2020). Thus, Mn_3_O_4_ nanoparticles could be a good candidate for environmental-friendly nanomaterials, and could be used to substitute CeO_2_ nanoparticles to improve plant salt tolerance. CeO_2_ nanoparticles improved rapeseed salt tolerance via ROS scavenging (Khan et al., 2022, 2021; Li et al., 2022a, b), shortening root apoplastic barrier (Rossi et al., 2017), modulation on plant hormones (Khan et al., 2022), and increase of α-amylase activities (Khan et al., 2021). To test whether Mn_3_O_4_ nanoparticles can be used to substitute CeO_2_ nanoparticles to improve plant salt tolerance, comparison of its effect on salt stressed plants between Mn_3_O_4_ and CeO_2_ nanoparticles should be conducted. Also, investigating the possible shared mechanisms in nanomaterials improved salt tolerance could give clues to design more efficient nanomaterials for agriculture. In this work, besides maintenance of ROS homeostasis, we tried to investigate the possible commonly shared mechanisms related to the maintenance of K^+^/Na^+^ ratio in rapeseed treated with Mn_3_O_4_ or CeO_2_ nanoparticles under salinity stress. K^+^ retention, Na^+^ exclusion, and vacuolar Na^+^ sequestration, which are the main mechanisms related to maintain K^+^/Na^+^ ratio (Munns and Tester, 2008; Li et al., 2022b), might be shared mechanisms between Mn_3_O_4_ and CeO_2_ nanoparticles improved salt tolerance in rapeseed.

In this study, we synthesized and characterized CeO_2_ and Mn_3_O_4_ nanoparticles. Whether CeO_2_ and Mn_3_O_4_ nanoparticles can improve rapeseed salt tolerance or not is investigated. Then, via confocal imaging and histochemical staining, we compared the ROS level of salt stressed rapeseed plants treated by CeO_2_ or Mn_3_O_4_ nanoparticles. Further, possible changes of Na^+^ and K^+^ content in salt stressed rapeseed plants treated by CeO_2_ or Mn_3_O_4_ nanoparticles were studied. Subcellular Na^+^ and K^+^ distribution was illustrated with Na^+^ and K^+^ dyes via confocal imaging techniques. Leaf total Na^+^ and K^+^ content was estimated by flame photometer. qPCR was conducted to investigate the changes of relative expression level of Na^+^ and K^+^ transport genes in salt stressed rapeseed plants treated by CeO_2_ or Mn_3_O_4_ nanoparticles. Overall, this study tried to investigate the possible commonly shared mechanism underlying CeO_2_ and Mn_3_O_4_ nanoparticles improved plant salt tolerance. Our results showed besides maintaining ROS homeostasis, the improvement on K^+^ retention ability is another shared mechanism between CeO_2_ and Mn_3_O_4_ nanoparticles improved rapeseed salt tolerance.

## Results

### Characterization and in vivo imaging of PNC and PMO

**Fig. 1:**
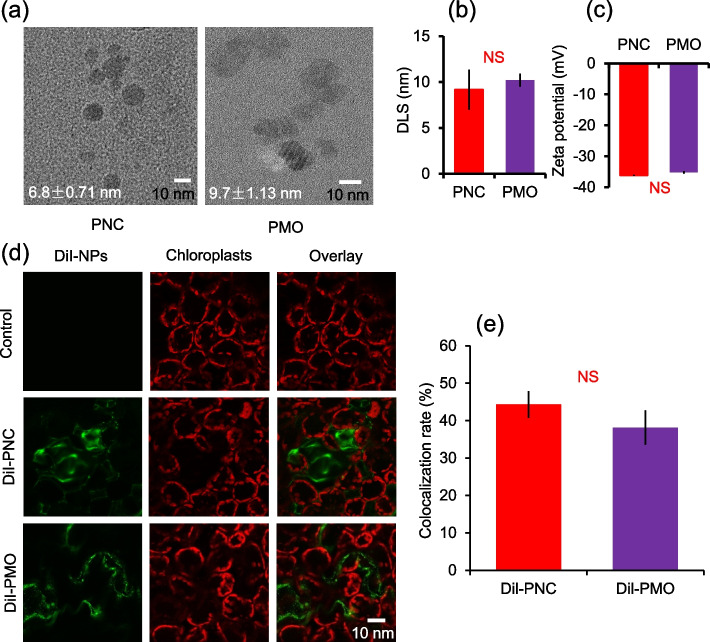
PNC and PMO characterization and subcellular distribution imaging in rapeseed leaf cell. **a** TME images of PNC (6.8 ± 0.71 nm) and PMO (9.7 ± 1.13 nm). **b** and **c** The hydrodynamic size and zeta potential of PNC and PMO. **d** Confocal images showed colocalization of chloroplasts autofluorescence with PNC or PMO. PNC and PMO were labelled with DiI fluorescent dye. **e** The colocalization rate between chloroplasts autofluorescence and DiI-PNC/DiI-PMO. Scale bar is 15 μm. Mean ± SE (*n* = 4–6). NS means no significant difference

TEM images showed that both PNC and PMO are spherical (Fig. 1a). The averaged TEM size of PNC and PMO are 6.8 ± 0.7 nm and 9.7 ± 1.1 nm, respectively (Fig. 1a). Dynamic light scattering (DLS) results showed that the size of PNC and PMO are 9.2 ± 2.2 nm and 10.2 ± 0.7 nm, respectively (Fig. 1b). The zeta-potential characterization (NanoBrook90 Plus) confirmed the negative charge of PNC and PMO (-36.2 ± 0.2 mV and -35.2 ± 0.4 mV) (Fig. 1c). The UV–Vis spectrum showed that PNC but not PMO have an absorption peak at 271 nm (Fig. S1a and c).

PNC and PMO were labelled with fluorescent dye 1,1′ -dioctadecyl-3,3,3′,3-tetramethylindocarbocyanine perchlorate (DiI) to allow its in vivo tracking (Fig. S1b and d). Both DiI-PNC and DiI-PMO have two peaks of absorbance at 517 nm and 557 nm, indicating the successful coating of DiI to PNC or PMO (Fig. S1a and c). Confocal imaging results showed that the colocalization rate between DiI-PNC/DiI-PMO and chloroplasts in the second true leaf of rapeseed are 44.3 ± 3.6% and 38.2 ± 4.6%, respectively (Fig. 1d and e). No DiI signal was found in the leaves of rapeseed treated with control buffer (Fig. 1d).

### PNC and PMO enhanced salt tolerance in salt stressed rapeseed plants

**Fig. 2:**
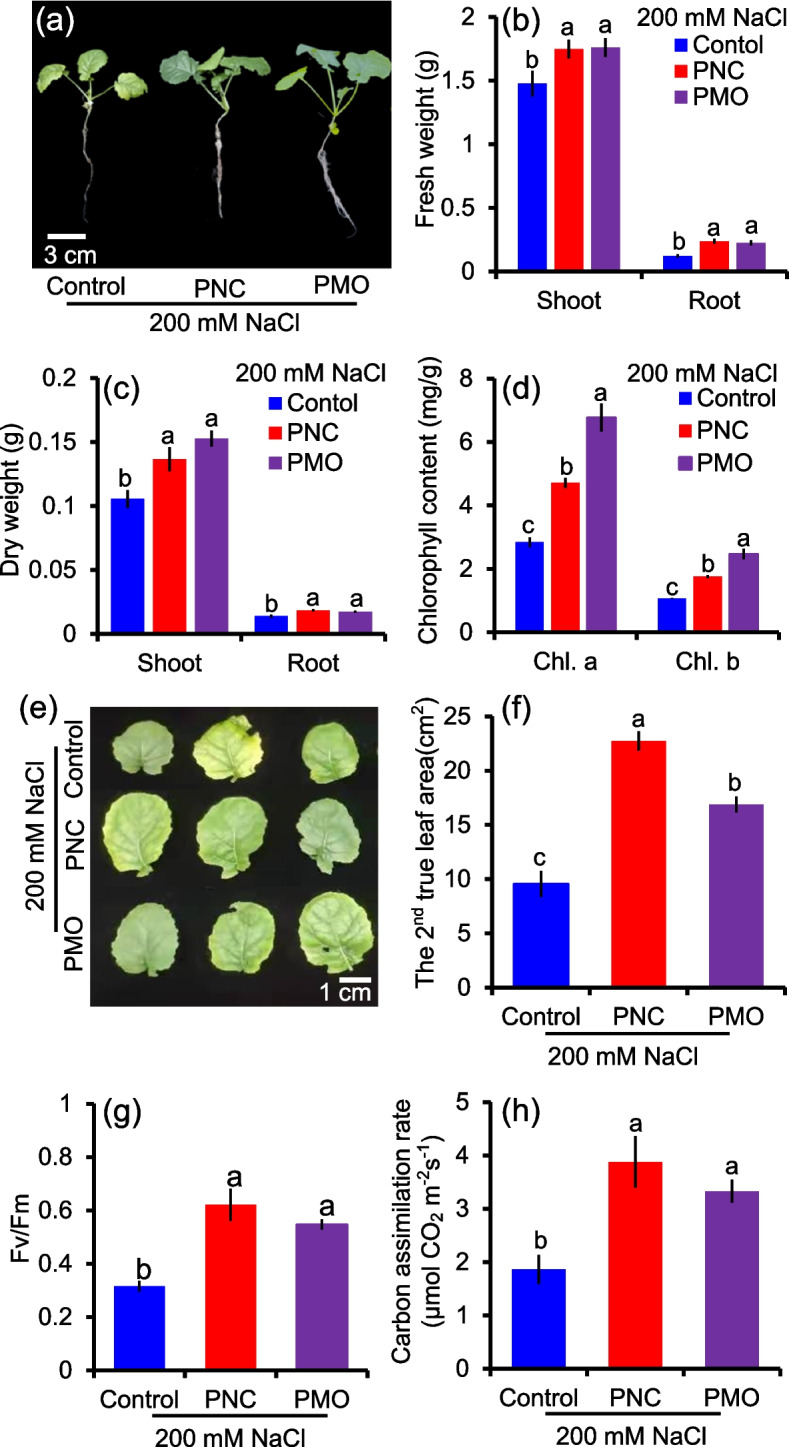
PNC and PMO enhanced salt tolerance in rapeseed plants after 12 days of 200 mM NaCl stress. **a** Phenotypic performance of salt stressed rapeseed plants with control buffer, PNC or PMO. **b** and **c** The biomass of rapeseed leaf and root treated with control buffer, PNC or PMO under salt stress. Mean ± SE (*n* = 18). **d** The chlorophyll content of the 2^nd^ true leaf of rapeseed treated with control buffer, PNC or PMO under salt stress. **e** and **f** Leaf image and area of the 2^nd^ true leaf of rapeseed treated with control buffer, PNC or PMO under salt stress. **g** and **h** Maximum yields of PSII (*Fv/Fm*) (**g**) and carbon assimilation rate (**h**) of the 2^nd^ true leaf of rapeseed treated with control buffer, PNC or PMO under salt stress. Different lowercase letters represent significance at 0.05 level. Mean ± SE (*n* = 6)

After 12 days of salt stress, plants treated with PNC or PMO showed better growth status compared with plants treated with control buffer (Fig. 2a). Compared with control treatment, under 200 mM NaCl, PNC increased 18.4% (1.75 ± 0.07 vs 1.48 ± 0.10) fresh weight and 29.5% (0.14 ± 0.01 vs 0.11 ± 0.006) dry weight in rapeseed shoot, and 96.5% (0.24 ± 0.02 vs 0.12 ± 0.01) fresh weight and 33.4% (0.02 ± 0.001 vs ± 0.01 ± 0.001) dry weight in rapeseed root, respectively (Fig. 2b and c). Similarly, compared with control plants under salt stress, PMO treated rapeseed plants have significant higher fresh weight (1.76 ± 0.07 vs 1.48 ± 0.10, 19.2% in shoot; 0.23 ± 0.02 vs 0.12 ± 0.01, 86.2% in root) and dry weight (0.15 ± 0.006 vs 0.11 ± 0.006, 44.7% in shoot; 0.02 ± 0.001 vs 0.01 ± 0.001, 25.5% in root) (Fig. 2b and c). Rapeseed leaves treated with PNC showed significantly higher chlorophyll content (chl. a, 4.7 ± 0.2 vs 2.8 ± 0.2; chl. b, 1.8 ± 0.1 vs 1.0 ± 0.1) than leaves treated with control buffer under salt stress (Fig. 2d). Compared with control under salt stress, PMO treated rapeseed plants also have higher chlorophyll content (chl.a, 6.8 ± 0.5 vs 2.8 ± 0.2; chl. b, 2.5 ± 0.2 vs 1.0 ± 0.1) (Fig. 2d). Similarly, compared with control group under salt stress, PNC and PMO treatment significantly increased 57.9% (22.7 ± 0.9 vs 9.6 ± 1.2) and 43.3% (16.9 ± 0.72 vs 9.6 ± 1.20) leaf area (the 2^nd^ true leaf), respectively (Fig. 2e and f). After 12 days of 200 mM NaCl stress, no significant difference in growth status, fresh weight of whole plant and the 2^nd^ true leaf area were found among CeCl_3_, MnCl_2_ and control treatments (Fig. S2a-c). Rapeseed plants treated with PNC or PMO exhibited higher *Fv/Fm* value (0.6 ± 0.06 vs 0.3 ± 0.02 or 0.5 ± 0.02 vs 0.3 ± 0.02) and carbon assimilation rate (3.9 ± 0.5 vs 1.9 ± 0.3 or 3.3 ± 0.2 vs 1.9 ± 0.3) than plants treated with control buffer, respectively (Fig. 2g and h). Moreover, to evaluate the biocompatibility between nanomaterials and rapeseed plants, we measured the effects of PNC and PMO on the growth of rapeseed seedlings under non-salt stress. No significant difference was found in growth status, chlorophyll content and fresh weight among all treatments (Fig. S4a-c).

### PNC and PMO reduced leaf ROS content in salt stressed rapeseed plants

**Fig. 3:**
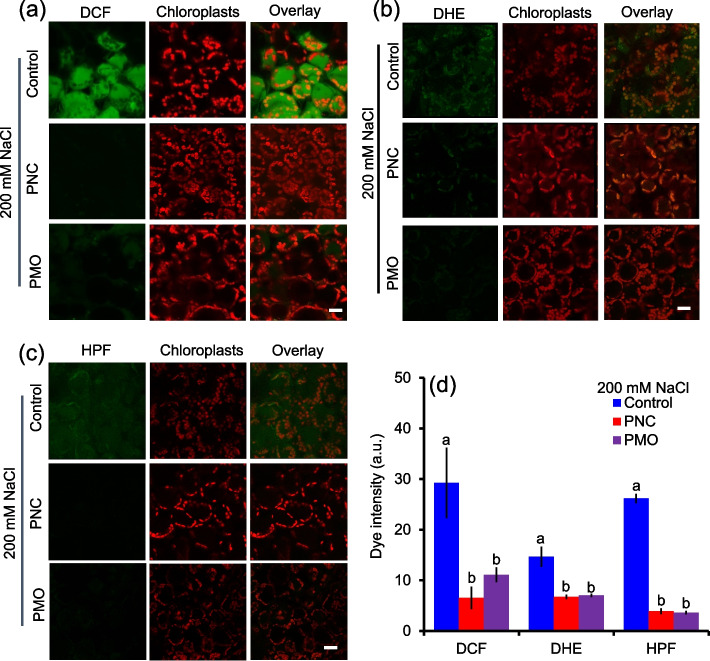
Confocal imaging of ROS fluorescent dye in rapeseed leaf after 12 days of 200 mM NaCl. **a**-**c** Confocal images of DCF dye (indicator of H_2_O_2_) (**a**), DHE dye (indicator of O_2_^• —^) (**b**) and HPF dye (indicator of OH^•^) (**c**) in mesophyll cells of rapeseed treated with control buffer, PNC or PMO under salt stress. (**d**) The calculated fluorescence intensity of DCF, DHE and HPF. Scale bar is 15 μm. Different lowercase letters represent significance at 0.05 level. Mean ± SE (*n* = 4–6)

**Fig. 4:**
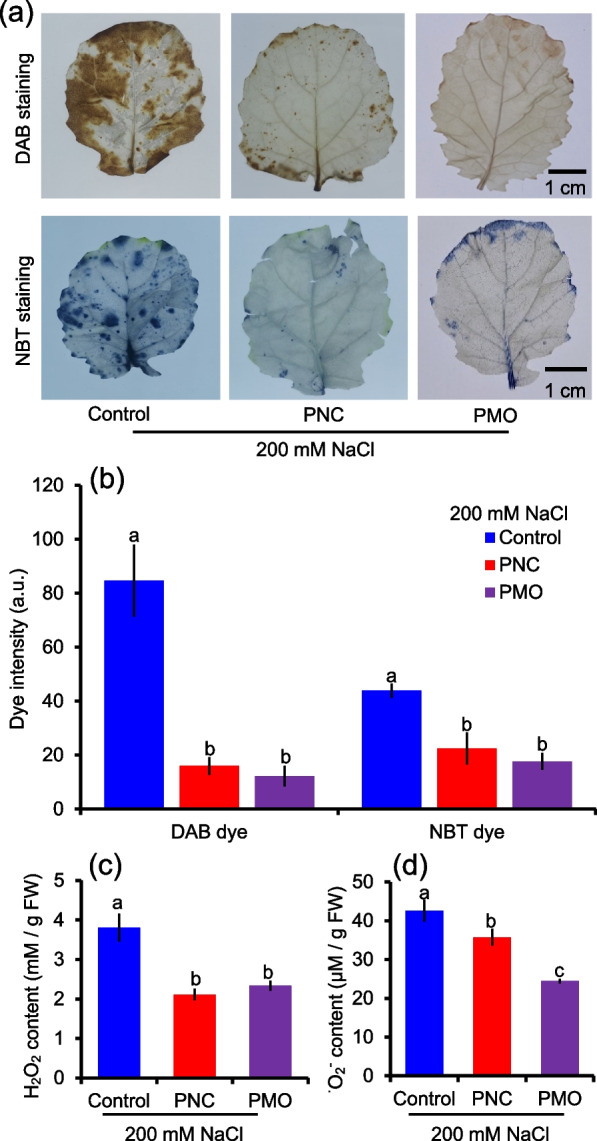
Histochemical staining and ROS content of rapeseed leaf under 200 mM NaCl stress. **a** DAB (3,3′-diaminobenzidine, for H_2_O_2_, dark brown spots) and NBT (nitro blue tetrazolium, for O_2_^• —^, blue spots) staining of leaves from salt stressed rapeseed treated with control, PNC or PMO. **b** The dye intensity of DAB and NBT were calculated by Image J software. **c** and **d** H_2_O_2_ and O_2_^•^^—^ content from salt stressed leaves of rapeseed treated with control buffer, PNC or PMO. Different lowercase letters represent significance at 0.05 level. Mean ± SE (*n* = 4–8)

As shown in confocal images, compared with control group, PNC and PMO treatment significantly decreased the fluorescence intensity of DCF (H_2_DCFDA, indicating H_2_O_2_), DHE (dihydroethidium, indicating O_2_• —) and HPF (Hydroxyphenyl fluorescein, indicating •OH) in salt stress rapeseed plants (Fig. 3a-c). Compared with control group under salt stress, PNC treated rapeseed plants showed significantly reduced intensity of DCF (6.5 ± 2.0 vs 29.3 ± 7.0, 77.7%), DHE (6.7 ± 0.5 vs 14.7 ± 2.2, 54.1%), and HPF dyes (3.9 ± 0.4 vs 26.2 ± 1.5, 85%) (Fig. 3d). Also, significantly lower ROS fluorescent dye intensity was showed in PMO treated rapeseed plants than control plants under salt stress, showing 62.1% (11.1 ± 0.9 vs 29.3 ± 7.0), 52.1% (7.0 ± 0.6 vs 14.7 ± 2.2) and 86% (3.6 ± 0.3 vs 26.2 ± 1.5) decrease in DCF, DHE and HPF intensity, respectively (Fig. 3d). Similarly, after 12 days of salt stress, PNC or PMO treated rapeseed plants showed significantly lower (2.1 ± 0.2 vs 3.8 ± 0.4, 44.5% decrease in PNC group; 2.3 ± 0.1 vs 3.8 ± 0.4, 38.6% decrease in PMO group) H_2_O_2_ content than control buffer treated plants (Fig. 4c). Also, compared with control, PNC and PMO group have 16.1% (35.8 ± 2.2 vs 42.6 ± 2.8) and 42.6% (24.5 ± 0.6 vs 42.6 ± 2.8) decrease of O_2_^• —^ content in rapeseed leaf under salt stress, respectively (Fig. 4d). No significant difference in leaf ROS content was found among CeCl_3_, MnCl_2_ and buffer treated rapeseed plants under salt stress (Fig. S3c and d).

This is further confirmed by histochemical staining experiments. Compared with control group, rapeseed leaves treated with PNC or PMO have significantly fewer brown spots (DAB dye) and blue dots (NBT dye) under salt stress (200 mM NaCl, 12 days) (Fig. 4a). After salt stress, leaves treated with PNC or PMO have significantly lower DAB (15.9 ± 3.3 vs 84.7 ± 13.4 and 12.2 ± 3.9 vs 84.7 ± 13.4) and NBT (22.4 ± 5.9 vs 43.9 ± 2.6 and 17.65 ± 3.2 vs 43.9 ± 2.6) intensity compared with leaves treated with control buffer (Fig. 4b). Similarly, no significant difference in leaf DAB and NBT staining and its intensity was found among CeCl_3_, MnCl_2_ and buffer treated rapeseed plants under salt stress (Fig. S3a and b).

### PNC and PMO maintained Na^+^/K^+^ ratio in salt stressed rapeseed plants

**Fig. 5:**
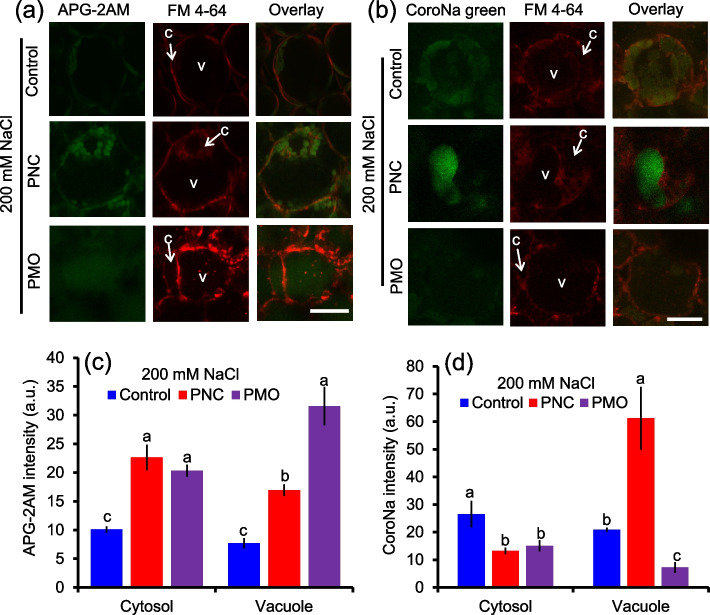
Confocal imaging of K^+^ and Na^+^ fluorescent dye in salt stressed (200 mM NaCl, 12 days) rapeseed leaves. **a** and **b**) Confocal images of APG-2AM dye (indicator of K^+^) (**a**) and CoroNa green dye (indicator of Na^+^) (**b**) in mesophyll cells of rapeseed treated with control buffer, PNC or PMO under salt stress. (c and d) The quantified analysis of APG-2AM (**c**) and CoroNa green (**d**) fluorescent dye in the vacuole and cytosol of mesophyll cells of rapeseed leaves from different treatments under salt stress. C means the cytosol and V means the vacuole. Scale bar is 15 μm. Different lowercase letters represent significance at 0.05 level. Mean ± SE (*n* = 4–6)

**Fig. 6:**
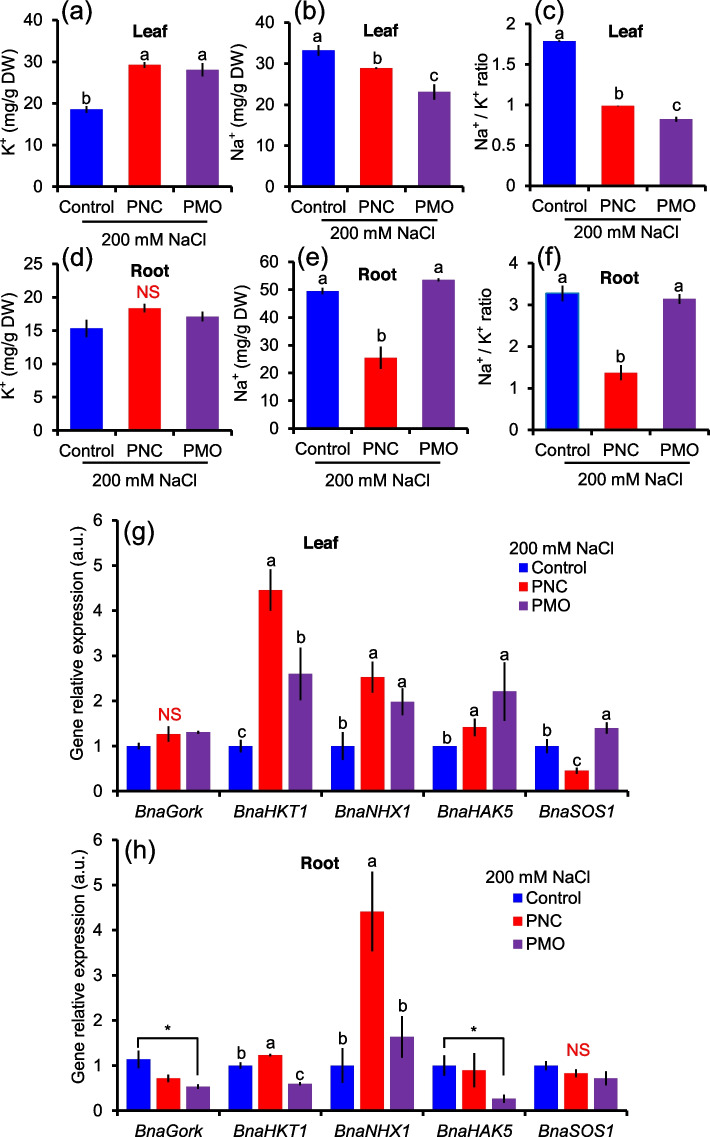
K^+^ and Na^+^ content and the relative expression level of K^+^ and Na^+^ transport related genes in salt stressed rapeseed plants. (**a**-**c**) K^+^ (**a**) and Na^+^ (**b**) content and Na^+^/K^+^ ratio (**c**) in the leaves of rapeseed treated with control buffer, PNC or PMO under salt stress. Mean ± SE (*n* = 8). (d-f) K^+^ (**d**) and Na^+^ (**e**) content and Na^+^/K^+^ ratio (**f**) in the roots of rapeseed treated with control buffer, PNC or PMO under salt stress. Mean ± SE (*n* = 8) (g and h) The relative expression level of K^+^ and Na^+^ transport related genes in the leaves (**g**) and roots (**h**) of rapeseed treated with control buffer, PNC or PMO under salt stress. Mean ± SE (*n* = 4). Different lowercase letters represent significance at 0.05 level. NS means no significant difference

After 12 days of salt stress, leaves of rapeseed treated with PNC or PMO have significantly higher APG-2AM (K^+^ fluorescent dye) intensity in the cytosol (22.7 ± 2.2 vs 10.1 ± 0.5 in PNC group; 20.3 ± 1.0 vs 10.1 ± 0.5 in PMO group) and the vacuole (16.9 ± 1.0 vs 7.7 ± 0.9 in PNC group; 31.58 ± 3.5 vs 7.7 ± 0.9 in PMO group) of mesophyll cells than the one treated with control buffer (Fig. 5a and c). Also, PNC and PMO group have significantly lower CoroNa green (Na^+^ fluorescent dye) intensity in the cytosol (13.3 ± 1.2 vs 26.6 ± 4.8 and 15.1 ± 2.1 vs 26.6 ± 4.8) of mesophyll cells than control group under salt stress (Fig. 5b and d). In the vacuole of mesophyll cells, PMO group also has lower CoroNa green intensity than control group (7.2 ± 1.9 vs 20.9 ± 0.8) under salt stress (Fig. 5b and d). On the contrary, PNC increased 65.9% (61.2 ± 11.4 vs 20.9 ± 0.8) CoroNa green intensity in the vacuole of mesophyll cells compared with control under salt stress (Fig. 5b and d). After 12 days of salt stress, PNC and PMO increased 36.4% (29.2 ± 0.7 vs 18.6 ± 0.8) and 33.8% (28.1 ± 1.6 vs 18.6 ± 0.8) K^+^ content and decreased 13.1% (28.9 ± 0.2 vs 33.2 ± 1.32) and 30.5% (23.1 ± 1.9 vs 33.2 ± 1.3) Na^+^ content in rapeseed leaf compared with control treatment, respectively (Fig. 6a and b). Moreover, PNC and PMO maintained lower Na^+^/K^+^ ratio (0.99 ± 0.03 vs 1.79 ± 0.01 and 0.82 ± 0.001 vs 1.79 ± 0.01, respectively) than control under salt stress (Fig. 6c). In rapeseed root, under salt stress, both PNC and PMO have no significant difference in K^+^ content of rapeseed root compared with control under salt stress (Fig. 6d). While, PNC but not PMO showed decreased Na^+^ content (25.48 ± 4.02 vs 49.51 ± 1.15, 48.5%) and Na^+^/K^+^ ratio (1.37 ± 0.18 vs 3.28 ± 0.19, 58.2%) compared with control group under salt stress (Fig. 6e and f).

### PNC and PMO modulated Na^+^ and K^+^ transport related genes in salt stressed rapeseed plants

Under 200 mM NaCl stress, no significant difference of relative gene expression level of *BnaGork* (gated outwardly rectifying K^+^ channel) was observed in rapeseed leaf treated with PNC, PMO or control buffer (Fig. 6g). Compare with control group under salt stress, the gene relative expression level of *BnaHKT1* (high affinity K^+^ transporter for Na^+^ exclusion), *BnaNHX1* (Na^+^/H^+^ exchanger for vacuolar Na^+^ sequestration) and *BnaHAK5* (K^+^ influx symporter) in rapeseed leaf were significantly upregulated in both PNC and PMO group (Fig. 6g). PMO significantly upregulated *BnaSOS1* (salt overly sensitive 1, Na^+^/H^+^ antiporter for Na^+^ exclusion) gene relative expression level in rapeseed leaf, whereas PNC significantly downregulated its expression compared with the control under salt stress (Fig. 6d). In rapeseed root, under salt stress, PMO but not PNC downregulated gene expression level of *BnaGork* and *BnaHAK5* compared with control (Fig. 6h). PNC upregulated gene expression level of *BnaHKT1* and *BnaNHX1* in rapeseed root compared with control group under salt stress (Fig. 6h). While, PMO treated rapeseed roots have significant lower gene expression level of *BnaHKT1* compared with buffer control treated rapeseed root under salt stress (Fig. 6h). Both PNC and PMO have no significant difference on *BnaSOS1* gene expression level in rapeseed roots compared with control group under salt stress (Fig. 6h).

## Discussion

### Mn_3_O_4_ nanoparticles are better than CeO_2_ nanoparticles in terms of improving plant salt tolerance

To feed over 9.3 billion projected populations in 2050, food production was estimated to be increased by 60% at 2005–2007 level (van Ittersum et al., 2016; Zhao et al., 2020). While, efficient agricultural production was affected by many factors, including biotic and abiotic stress (Anzano et al., 2022). For abiotic stress, salinity is one of the main factors limiting efficient agriculture production (Wu et al., 2018a). To cope with salinity issue, many approaches were tried, ranging from irrigation (including flushing saline soil with fresh water) (Shrivastava and Kumar, 2015), phytoremediation (Barassi et al., 2006; Imadi et al., 2016), to breeding salt tolerant crop varieties (Ylvisaker, 1982). However, phytoremediation and breeding program take long time. Irrigation, especially flushing saline soil with fresh water, is not a sustainable way in semi-arid area. To address salinity issue in a short production period, new solutions which can help to improve crop salt tolerance are encouraged.

Here, our results showed that both PNC and PMO increased rapeseed salt tolerance (Fig. 2a). This is in accordance with previous studies showed that nanoceria improved salt tolerance in rice (Zhou et al., 2021), cotton (An et al., 2020; Liu et al., 2021), rapeseed (Khan et al., 2022, 2021; Rossi et al., 2016, 2017), cucumber (Chen et al., 2022), Arabidopsis (Wu et al., 2018a) and Moldavian Balm (Mohammadi et al., 2021). Unlike applications of nanoceria in many plant species, to date, Mn_3_O_4_ nanoparticles were only used in cucumber to improve its salt tolerance (Lu et al., 2020). Here, in this study, we showed that foliar applied PMO enabled better salt tolerance in rapeseed than PNC treatment, showing that PMO treated rapeseed plants have significant higher chlorophyll content (Fig. 2d), ROS homeostasis (Fig. 4d) and K^+^/Na^+^ ratio (Fig. 6c) than PNC treated rapeseed plants under salinity. Moreover, as mentioned early, compared with heavy metal concern of cerium, Mn is an essential plant nutrient and Mn fertilizer is widely used in agriculture production (Deng et al., 2020). Together, it suggests that PMO are a better candidate than PNC for nano-improved plant salt tolerance.

Moreover, it should be noted that compared with control plants, rapeseed treated with either PNC or PMO showed significant better root performance under salinity stress (Fig. 2a). This is in consistent with previous studies, which showed that rapeseed primed with PNC treated plants showed much better root performance than control group under salt stress (An et al., 2020; Khan et al., 2021, 2022). Moreover, it should be noted that although CeCl_3_ and MnCl_2_ treatment also improved rapeseed root performance than control plants under salt stress, no significant difference of whole plant fresh weight was found among these treatment groups (Figure S2a). These results suggest that unlike nano-enabled improvement at whole plant level, inorganic salt control only have effects on some organs/tissues and does not improve stress performance at whole plant level. The behind mechanisms are worthy to be studied in future studies.

### Improvement of leaf K^+^ retention and maintaining ROS homeostasis are commonly shared mechanisms between CeO_2_ and Mn_3_O_4_ nanoparticles improved salt tolerance

ROS are a double sword for plants. High level ROS are toxic to plant cells, while low level ROS are signals (Mittler, 2017). To maintain ROS homeostasis, plants evolved both enzymatic (including SOD, POD, CAT and APX etc.) and non-enzymatic (including proline, GSH, ascorbic acid and flavonoids etc.) antioxidant system(You and Chan, 2015). Under salinity stress, ROS over-accumulation is a secondary stress in plants (Zhu, 2016). Thus, plants with better ability to maintain ROS homeostasis are always associated with stronger salt tolerance (Bose et al., 2014). This is also showed in nano-improved plant salt tolerance. In nano-improved plant salt tolerance, the improved phenotypic performance and increased of ROS scavenging ability (or decreased ROS level) are always observed simultaneously in many plant species, such as rapeseed (Khan et al., 2022, 2021; Li et al., 2022a, b; Rossi et al., 2016, 2017), rice (Zhou et al., 2021), wheat (Saad-Allah and Ragab, 2020), maize (Liu et al., 2022), cotton (An et al., 2020; Liu et al., 2021), cucumber (Chen et al., 2022; Lu et al., 2020), grapevine (Gohari et al., 2021) and Arabidopsis (Wu et al., 2018a). For example, CeO_2_ nanoparticles significantly decreased the ROS content of rapeseed leaf under 200 mM NaCl stress (Li et al., 2022a, b). Also, sulfur nanoparticles increased of ROS scavenging ability of wheat under salt stress (Saad-Allah and Ragab, 2020). Thus, maintaining ROS homeostasis could be a commonly employed mechanism underlying nano-improved plants salt tolerance.

K^+^ is an essential nutrient for plants. K^+^ plays important role in plant activities, such as stomatal movement (Wu et al., 2018a), enzyme activation (Wu et al., 2018b), and turgor pressure (Whatmore and Reed, 1990) etc. K^+^ retention is a known mechanism related to plant salt tolerance. For example, salt tolerant barley varieties always showed higher root (Chen et al., 2005) and mesophyll (Wu et al., 2013, 2015) K^+^ retention ability than the salt sensitive one. Similar results were found in other plant species, such as wheat (Cuin et al., 2008; Wu et al., 2014), rice (Liu et al., 2019) and poplar trees (Sun et al., 2009). Indeed, this is also observed in nano-improved plant salt tolerance. Previous study showed that compared with salt stressed control plants, ROS scavenger nanoceria helped to retain mesophyll K^+^ in Arabidopsis plants under salinity stress (Wu et al., 2018a). Nanoceria improved leaf K^+^ retention in cotton plants under salt stress (Liu et al., 2021). Also, significant higher leaf K^+^ content was found in Mn_3_O_4_ nanoparticles treated cucumber plants than control plants under salinity stress (Lu et al., 2020). Furthermore, Na^+^ content and Na^+^/K^+^ ratio were decreased and K^+^ content was increased in pearl millet by seed priming with silver nanoparticles (Khan et al., 2020). Gold nanoparticles was reported that it improved the K^+^ concentration and turn positively affected the stomatal traits in wheat under salt stress (Wahid et al., 2022). Here, our results showed that both PNC and PMO enabled better leaf K^+^ retention in salt stressed rapeseed plants (Fig. 5a and c, Fig. 6a). Together, it further suggests that improvement of K^+^ retention could be a commonly shared mechanism in nano-improved plant salt tolerance.

Furthermore, plant’s ability to maintain K^+^ under salinity stress is known to be associated with its ability to scavenging ROS. K^+^ efflux channels such as *GORK* and ROS-activated NSCC channels can be stimulated by ROS, resulting in massive K^+^ loss (Demidchik, 2014; Wu et al., 2018a). Thus, maintaining ROS homeostasis could be helpful to retain K^+^ in plants under salinity stress. As mentioned above, maintaining ROS homeostasis could be commonly employed for nano-improve plant salt tolerance. Thus, again, improvement of K^+^ retention ability might subsequently be a common mechanism for nano-improved plant salt tolerance. Moreover, compared with no significant change of relative expression level of *BnaGORK*, *BnaHAK5* gene was significantly upregulated in rapeseed plants treated with either PNC or PMO under salinity (Fig. 6g). This is in accordance with previous study showing that nanoceria treated Arabidopsis showed significantly upregulation of *HAK5* gene, while no change on *GORK* gene (Wu et al., 2018a). Giving the fact that K^+^ efflux was reduced and *HAK5* mediated K^+^ uptake requires ATP consumption, nanoparticles (i.e. PNC and PMO in this study) might be directly interact with K^+^ efflux channels to modulate its activities.

### Different mechanisms are employed to maintain cytosolic Na^+^ homeostasis between CeO_2_ and Mn_3_O_4_ nanoparticles improved salt tolerance

Cytosolic K^+^/Na^+^ ratio is a hallmark for plant salt tolerance (Liu et al., 2021). Besides K^+^ retention, removal of excessive Na^+^ in cytosol is also of importance for maintain cytosolic K^+^/Na^+^ ratio homeostasis. Under salinity stress, massive entry of Na^+^ not only can compete with K^+^ for the binding to enzymes, but also can cause rapid membrane depolarization and impose osmotic pressure (Shabala and Munns, 2017). Na^+^ exclusion and vacuolar Na^+^ sequestration are two known mechanisms to remove cytosolic Na^+^ (Carden et al., 2003; Wu et al., 2019). Here, in this study, we found that both PNC and PMO treated rapeseed plants showed significantly lower leaf Na^+^ content and Na^+^/K^+^ ratio than control plants under salt stress (Fig. 6b and c). This is in accordance with previous results showing less Na^+^ over-accumulation in nanoceria treated plants i.e. Arabidopsis, cotton and rapeseed under salt stress (Liu et al., 2021; Wu et al., 2018a). Further analysis showed that both PNC and PMO treated rapeseed plants have lower cytosolic Na^+^ signals than control plants under salt stress. While, compared with control plants, PNC and PMO treated rapeseed plants respectively showed significantly higher and lower vacuolar Na^+^ signals (Fig. 5d). It suggests that compared with only increased Na^+^ exclusion ability in PMO treated rapeseed plants, PNC treated plants showed stronger Na^+^ exclusion and vacuolar Na^+^ sequestration than control plants under salt stress. It showed that PNC and PMO employed different mechanisms to maintain cytosolic Na^+^ homeostasis. Furthermore, previous studies showed that in cotton, PNC improved shoot Na^+^ exclusion but not vacuolar Na^+^ sequestration (Liu et al., 2021). While, in cucumber plants, Mn_3_O_4_ nanoparticles did not change Na^+^ level in leaf, stem and root under salt stress (Lu et al., 2020). It suggests the complexity of mechanisms underlying nano-improved plant salt tolerance.

Moreover, although PNC and PMO treated rapeseed plants showed significantly lower cytosolic Na^+^ and leaf Na^+^ than control plants under salinity stress, the relative expression level of *BnaSOS1* gene is respectively down-regulated and upregulated (Fig. 6g). It suggests the employed mechanisms to maintain low cytosolic Na^+^ in PNC and PMO treated rapeseed plants are different. Indeed, PNC treated rapeseed plants showed higher upregulation of *BnaHKT1* and *BnaNHX1* genes than PMO treated rapeseed plants under salt stress (Fig. 6d). This could partially explain that with down-regulation of *BnaSOS1*, PNC treated rapeseed plants can maintain lower cytosolic Na^+^ than control plants under salt stress. Previous studies also showed that *SOS1* gene expression pattern can be different in nano-improved plant salt tolerance. In cotton, foliar application of nanoceria did not alter the expression level of *SOS1* and *NHX1* genes but upregulated *HKT1* gene in leaf cells under salt stress (Liu et al., 2021). Under salt stress, iron oxide nanoparticles treated *Eucalyptus tereticornis* Sm. plants showed significant upregulation of *SOS1, NHX1* and *HKT1* genes than control plants (Singh et al., 2021). Moreover, under salt stress, the expression profile of Na^+^ and K^+^ transport related genes was varied in root and shoot of rapeseed treated with PNC and PMO (Fig. 6g and h). Overall, it suggests that the mechanisms employed to maintain cytosolic Na^+^ in nano-improved plant salt tolerance can be varied significantly in plant species with different nanomaterials, even at plant tissue level.

## Materials and methods

### Synthesis and characterization of PNC and PMO

Poly(acrylic) acid coated cerium oxide nanoparticles (PAA@CeO_2_-NPs, PNC) were synthesized in lab as described in our previous papers (Li et al., 2022a, b; Wu et al., 2017). Briefly, 1.08 g of cerium (III) nitrate (Sigma Aldrich, 99%) and 4.5 g poly (acrylic) acid (MW 1800, Sigma Aldrich) were dissolved in 2.5 mL and 5 mL ddH_2_O in 50 mL conical tube, respectively. Then, the solutions were mixed at 2, 000 rpm for 15 min via a vortex mixer (VORTEX-7). The mixture solution was added dropwise to 15 mL of 30% ammonium hydroxide solution (Sigma Aldrich) in a 50 mL glass beaker and stirred at 500 rpm overnight at ambient temperature. After 24 h, the final solution was centrifuged at 4,000 rpm for 1 h to remove debris and large agglomerates. Then, using a 10 K Amicon cell (MWCO 10 K, Millipore Inc.), 15 mL supernatant was purified from free polymers and other reagents by centrifugation at 4,500 rpm for 45 min. Centrifugation process was repeated at least six times. The purified PNC solution was filtered through a 100 nm pore size syringe filter (BIOFIL).

Poly(acrylic) acid coated manganese oxide nanoparticles (PAA@Mn_3_O_4_-NPs, PMO) were synthesized in lab using the following method. Briefly, 0.425 g MnSO_4_·H_2_O (Sigma Aldrich, 99%) and 4.5 g poly(acrylic) acid (MW 1800, Sigma Aldrich) were dissolved in 2.5 mL and 5 mL deionized water, respectively. Then, the solutions were mixed at 2, 000 rpm for 15 min via a vortex mixer (VORTEX-7). The mixture solution was added dropwise to 15 mL of 30% ammonium hydroxide solution (Sigma Aldrich) in a 50 mL glass beaker and stirred at 500 rpm overnight at ambient temperature. Then, the solution was added into a 50 mL Teflon-equipped stainless autoclave, with heating at 120 °C for 24 h. The brown solution was centrifuged at 4, 000 rpm for 1 h to remove any debris and large agglomerates. The supernatant was further purified by dialysis bag (MW 10 kD, Xi’an Yobios Biotechnology Co. ltd.) for 24 h. The water was refreshed once every 8 h. The purified PMO solution was filtered through a 100 nm pore size syringe filter (BIOFIL).

The final PNC and PMO solution were stored in 4 °C refrigerator for further use. PNC and PMO were characterized as described in our previous papers (Li et al., 2022a, b; Wu et al., 2017). Briefly, the hydrodynamic diameter (DLS size) and zeta potential of PNC and PMO were measured by a Brookhaven Zeta-sizer (NanoBrook90 Plus). TEM imaging of PNC and PMO were done by using an FEI Talos microscope operating at 300 kV. The UV–Vis spectrum of PNC and PMO were measured by a UV–Vis spectrophotometer (UV-1800, AOE).

### Plant materials and treatments

Seeds of rapeseed (*Brassica napus* L. Zhongshuang 11) (ZS11), were disinfected with 1% sodium hypochlorite for 15 min and then washed by running tap water for 30 min. After washing, seeds were germinated in plastic boxes (12 cm × 12 cm × 5 cm) with 3 layers of germination paper for 7 days. The uniform germinated seedlings were transplanted into small pots (7 cm × 7 cm × 8 cm) filled with mixture soil (substrate and vermiculite, 3:1, v: v). 12 small pots were transferred into a black tray (45 cm × 30 cm × 8 cm) and then 1.5 L water was added into each tray. After a week, 1 L, 1/2 Hoagland solution was added. 1 mL solution [buffer, 0.05 mM PNC (~ 5.6 mg/L) or 300 mg/L PMO] was sprayed on the rapeseed leaf at the second leaf stage. After spraying, seedlings were transferred to low light condition (about 20 μmol m^−2^ s^−1^). After 3 h, 1.5 L, 200 mM NaCl solution was added into the black tray. 1 L, 200 mM NaCl solution was added weekly. The second true leaf was sampled for further experiment after 12 days of salt stress. Plant growth condition was described as our previous paper (Li et al., 2022a, b; Liu et al., 2021): 200 μmol m^−2^ s^−1^ photosynthetic active radiation (PAR), 24 ± 1 °C and 21 ± 1 °C in daytime and night, 60% relative humidity, and 14 h/10 h of day/night regime.

### PNC and PMO labelled with DiI fluorescent dye for confocal imaging

As described in previous papers (Newkirk et al., 2018), PNC and PMO were labelled with 1,1′-dioctadecyl-3,3,3′,3′-tetramethylindo-carbocyanine perchlorate (DiI) fluorescent dye to observe their distribution in rapeseed leaf cells. Briefly, 200 μL DiI dye solution (0.3 mg/L, in dimethyl sulfoxide) was added dropwise to 10 mL glass bottles contain 4 mL of 0.5 mM PNC or 900 mg/L PMO, then the mixture solutions were stirred at 1, 000 rpm for 1 min at ambient temperature. The resulting solutions (DiI-PNC and DiI- PMO) were transferred into 15 mL 10 kDa Amicon cell (MWCO 30 K, Milipore Inc.) and ddH_2_O was added to make the volume to 15 mL. Then, the solution was centrifuged at 4, 500 rpm in five cycles (5 min each cycle) to remove free DiI dye. Following our previous paper (Wu et al., 2017), the visualization of DiI-PNC and DiI-PMO in rapeseed leaf cells were performed by Leica spectral confocal laser scanning microscope (TCS-SP8, Leica Microsystems, Germany). Briefly, after 3 h incubation of the spray application of DiI-PNC or DiI-PMO in rapeseed leaves, samples (leaf discs, 5 mm of diameter) were taken with a cork borer and mounted on glass slides. Perfluorodecalin (PFD) was dropped to each slide to improve the quality of confocal imaging. A coverslip was placed on the leaf disc, ensuring that no air bubbles trapped underneath. The imaging settings are as follows: 40 × wet objective; 514 nm laser excitation; PMT1: 550 − 615 nm; PMT2: 700 − 800 nm. Colocalization analysis was performed in LAS (Leica Application Suit) AF Lite software following our previous publications (Li et al., 2022a, b; Wu et al., 2017). Four to eight biological replicates were used.

### Rapeseed plant performance under salinity stress

After 12 days of salt stress (200 mM NaCl), the phenotype images of rapeseed seedling were taken by a Canon 90D camera. The second true leaf was sampled for further experiments. Chlorophyll content was measured following our previous publication (Li et al., 2022a, b). Briefly, 0.1 g fresh leaf was soaked into 10 mL mixture solution containing acetone and ethanol (1:1, v: v) at dark condition on a shaker (50 rpm). After 24 h, solution was centrifuged at 2, 000 rpm for 10 min. Then, the supernatant was transferred to a 10 mL tube. The chlorophyll content was calculated by measuring the absorbance at 644 nm and 662 nm via UV–Vis spectrophotometer (UV-1800, AOE). Chlorophyll content was calculated using the following equations:

$$\mathrm{Chlorophylla}\;\mathrm{content}=9.784\;\times\;{\mathrm A}_{662}-0.99\;\times\;{\mathrm A}_{644}$$

$$\mathrm{Chlorophyllb}\;\mathrm{content}=21.426\;\times\;{\mathrm A}_{644}-4.65\;\times\;{\mathrm A}_{662}$$

where A_662_ and A_644_ are the absorbance value measured at 662 nm and 644 nm, respectively.

After 12 days of salt stress, rapeseed seedlings were sampled. The shoot part was collected as described elsewhere. For the root, the soil at rapeseed roots was carefully washed away by tap water. Fresh weight was measured immediately after finishing the sampling process. For the dry weight, rapeseed plants were dried at 105 °C for 30 min, and then dried at 85 °C for 3 days. The area of the second true leaf was analyzed by Image J software. Photosynthetic parameters (carbon assimilation rate and *Fv/Fm* value) of the second true leaf were measured by portable photosynthetic apparatus Li-6800. The measurement settings are: 1,000 μmol m^−2^ s^−1^ photosynthetic photon flux density, 400 μmol mol^−1^ CO_2_ concentration, and 25 °C leaf temperature.

### Measurement of leaf ROS content

As described in previous studies (Li et al., 2022a, b; Liu et al., 2021), the measurement of superoxide anion (O_2_^• —^) and hydrogen peroxide (H_2_O_2_) content were done by using the kits from “Solarbio Life Sciences (Item number: 20210903)” and “Nanjing Jiancheng Biotechnology Co., Ltd (item number: A04-1–1)”, respectively. The contents of H_2_O_2_ and O_2_^• —^ were calculated by the manuals from manufacturers.

### DAB and NBT staining

According to protocols in previous study (Kumar et al., 2013), DAB (3,3′-diaminobenzidine) and NBT (nitro blue tetrazolium) staining were performed. Briefly, 50 mg DAB and 100 mg NBT were dissolved in 45 mL ddH_2_O (adjust pH to 3.8 by 3 M HCl) and 50 mL 50 mM sodium phosphate buffer (pH 7.5), respectively. After 12 days of salt stress, the second true leaves of rapeseed plants were soaked in fresh staining solution overnight at dark and ambient temperature. After staining, leaves were washed with ddH_2_O. Then, leaf chlorophyll was removed with boiled mixture solution of ethanol and glycerin (9:1, v: v). Photos were taken with a Canon 90D camera.

### In vivo ROS scavenging by PNC and PMO

Confocal imaging of ROS level in leaves from rapeseed treated with PNC or PMO was performed as described in our pervious paper (Li et al., 2022a, b; Wu et al., 2018a). 25 μM H^2^DCFDA (2′,7′-dichlorodihydrofluorescein diacetate, Thermo Fisher Scientific, Lot No. D399), 10 μM DHE (dihydroethidium, Thermo Fisher Scientific, Lot No. D23107) and 10 μM HPF (2-[6-(4'-Hydroxy) phenoxy-3H-xanthen-3-on-9-yl]benzoicacid, Sigma Aldrich, Lot No. SLCG8850) fluorescent dye were used to visualize ROS in leaf tissues. Briefly, after 12 days of salt stress, leaf discs of rapeseed were incubated with H^2^DCFDA, DHE and HPF in 1.5 mL Eppendorf tubes for 30 min under darkness.. After incubation, the leaf discs were mounted on the glass slides. For better confocal imaging, a drop of PFD was dropped onto each slide. A coverslip was carefully put onto the mounted sample, ensuring that no air bubbles remained trapped. The samples were imaged by a Leica SP8 confocal microscope (Leica Microsystems, Germany). The imaging settings are as follows: 40 × wet objective; 488 nm laser excitation (30%); PMT1: 500–600 nm; PMT2: 700–800 nm. Four to eight biological replicates were used. The fluorescence intensity of H_2_DCFDA, DHE and HPF were calculated by LAS (Leica Application Suit) AF Lite software.

### The estimation of leaf Na^+^ and K^+^ content

0.1 g dry leaf or root samples were digested in a 50 mL digestion tube filled with 5 mL H_2_SO_4_ (18.4 M) by a microwave digester (LWY848, SiPing Electronic Research Institute, China). After digestion, the solution was diluted with ddH_2_O to 50 mL. The final solution was filtered through a 450 nm pore size syringe filter (BIOFIL). Flame photometer (FP6431, Jiangke, Shanghai, China) was used to determine the content of Na^+^ and K^+^.

### Confocal imaging of Na^+^ and K^+^ distribution in mesophyll cells

Confocal imaging of Na^+^ and K^+^ distribution in the cytosol and vacuole was performed using 20 μM CoroNa Green AM (Na^+^ dye, Thermo Fisher Scientific), 20 μM APG-2 AM (Asante potassium green-2AM, K^+^ dye, Abcam Biotechnology company) and 20 μM FM 4–64 (*N*-(3-triethykammoniumpropyl)-4(6(4-(diethylamino)phenyl)hexatrienyl)pyridinium dibromide, membrane dye, Thermo Fisher Scientific) following our previous publications (Liu et al., 2021). Briefly, CoroNa Green AM and APG-2 AM were dissolved in pure dimethyl sulfoxide (DMSO) and diluted with MES buffer (10 mM KCl, 5 mM Ca^2+^-MES, pH 6.1) and TES buffer (10 mM, pH 7.5) to working concentration, respectively. Under salinity stress (200 mM NaCl after 12 days), leaves of rapeseed treated with PNC or PMO were collected. The upper epidermis of leaves was gently peeled off to allow better incubation with fluorescent dyes. Leaf discs were incubated with mixture solution of CoroNa Green AM + FM 4–64 or APG-2 AM + FM 4–64 in 1.5 mL Eppendorf tubes for 2 h and 2.5 h in darkness, respectively. Leaf discs were mounted on the glass slides after incubation. For better confocal imaging, a drop of PFD was added. A square coverslip was carefully put onto the mounted sample to cover the sample completely. The imaging settings are as follows: 40 × wet objective; 488 nm laser excitation (30%); PMT1: 500–550 nm (for Na^+^ confocal imaging) or 520–560 nm (for K^+^ confocal imaging); PMT2: 610–630 nm (for membrane imaging). Four to eight biological replicates were used. The fluorescence intensity of CoroNa Green AM and APG-2 AM were calculated by image J software.

### RNA isolation and quantitative real-time PCR (qRT-PCR) analysis

The leaf and root RNA was extracted and synthesized to cDNA by using RNA prep Pure Plant Kit (RN38, Aidlab, Beijing, China) and TRUE-script first Strand cDNA Synthesis Kit (PC5402, Aidlab, Beijing, China) following the manufacturer’s instruction. Quantitative real-time PCR was performed by SYBR Green qPCR Mix (PC3302, Aidlab, Beijing, China). The relative expression level of studied genes was analyzed by 2^−ΔΔCT^ method (Livak and Schmittgen, 2001). The primers used for qRT-PCR were shown in Table S1.

### Statistical analysis

All data (mean ± SE, *n* = biological replicates) were analyzed using SPSS 23.0. Comparison between treatments was performed by independent samples *t*-test (two tailed) or one-way ANOVA based on Duncan’s multiple range test (two tailed). The significance levels were *P < 0.05, **P < 0.01, and ***P < 0.001. Different lowercase letters mean the significance at *P* < 0.05.

## Supplementary Information

**Additional file 1:Table S1.** Primers used for quantitative real-time PCR (qRT-PCR) analysis. **Figure S1.** PNC and PMO characterization**.** (a and c) The absorbance spectrum of PNC/DiI-PNC (a) and PMO/DiI-PMO (c). (b and d) The solution of final PNC/DiI-PNC (b) and PMO/DiI-PMO (d). **Figure S2.** Effect of CeCl_3_ and MnCl_2_ on rapeseed seedlings under salt stress. (a) The growth status of rapeseed seedlings treated with CeCl_3_ (0.05 mM), MnCl_2_ (300 mg/L) or control buffer after 12 days of 200 mM NaCl stress. (b and c) The 2^nd^ true leaf area (b) and whole plant fresh weight (c) of rapeseed treated with CeCl_3_, MnCl_2_ or control buffer after 12 days of 200 mM NaCl stress. Mean ± SE (*n*=4-15). NS means no significant difference.**Figure S3**. Histochemical staining and ROS content of rapeseed leaf under 200 mM NaCl stress. (a) DAB (for H_2_O_2_, dark brown spots) and NBT (for O_2_^•^^—^, blue spots) staining of leaves from salt stressed rapeseed treated with CeCl_3_ (0.05 mM), MnCl_2_ (300 mg/L) or control buffer. (b) The dye intensity of DAB and NBT were calculated by Image J software. (c and d) H_2_O_2_ and O_2_^•^^—^ content from salt stressed leaves of rapeseed treated with CeCl_3_ (0.05 mM), MnCl_2_ (300 mg/L) or control buffer. Mean ± SE (n=4-8). NS means no significant difference. **Figure S4.** Effects of PNC, PMO, CeCl_3_ and MnCl_2_ on rapeseed seedlings growth under non-stress conditions. (a) Phenotypic performance of rapeseed plants treated with different solution under non stress condition. (b) The chlorophyll content of the 2^nd^ true leaf of rapeseed treated with different solution under non stress condition. (c) The fresh weight of whole rapeseed plant treated with different solution under non stress condition. Mean ± SE (n=4-15). NS means no significant difference.

## Data Availability

The materials used in this study will be available for research upon reasonable request.
