# Supplementary material for: Improvement of leaf K+ retention is a shared mechanism behind CeO2 and Mn3O4 nanoparticles improved rapeseed salt tolerance
Source: Stress Biol. 2022 Nov 8;2(1):46. doi: 10.1007/s44154-022-00065-y (PMC10441935; doi:10.1007/s44154-022-00065-y)
Supplement: Supplementary file 1 — Additional file 1:Table S1. Primers used for quantitative real-time PCR (qRT-PCR) analysis. Figure S1. PNC and PMO characterization. (a and c) The absorbance spectrum of PNC/DiI-PNC (a) and PMO/DiI-PMO (c). (b and d) The solution of final PNC/DiI-PNC (b) and PMO/DiI-PMO (d). Figure S2. Effect of CeCl3 and MnCl2 on rapeseed seedlings under salt stress. (a) The growth status of rapeseed seedlings treated with CeCl3 (0.05 mM), MnCl2 (300 mg/L) or control buffer after 12 days of 200 mM NaCl stress. (b and c) The 2nd true leaf area (b) and whole plant fresh weight (c) of rapeseed treated with CeCl3, MnCl2 or control buffer after 12 days of 200 mM NaCl stress. Mean ± SE (n=4-15). NS means no significant difference.Figure S3. Histochemical staining and ROS content of rapeseed leaf under 200 mM NaCl stress. (a) DAB (for H2O2, dark brown spots) and NBT (for O2•—, blue spots) staining of leaves from salt stressed rapeseed treated with CeCl3 (0.05 mM), MnCl2 (300 mg/L) or control buffer. (b) The dye intensity of DAB and NBT were calculated by Image J software. (c and d) H2O2 and O2•— content from salt stressed leaves of rapeseed treated with CeCl3 (0.05 mM), MnCl2 (300 mg/L) or control buffer. Mean ± SE (n=4-8). NS means no significant difference. Figure S4. Effects of PNC, PMO, CeCl3 and MnCl2 on rapeseed seedlings growth under non-stress conditions. (a) Phenotypic performance of rapeseed plants treated with different solution under non stress condition. (b) The chlorophyll content of the 2nd true leaf of rapeseed treated with different solution under non stress condition. (c) The fresh weight of whole rapeseed plant treated with different solution under non stress condition. Mean ± SE (n=4-15). NS means no significant difference. [file 44154_2022_65_MOESM1_ESM.pptx]

## Slide 1
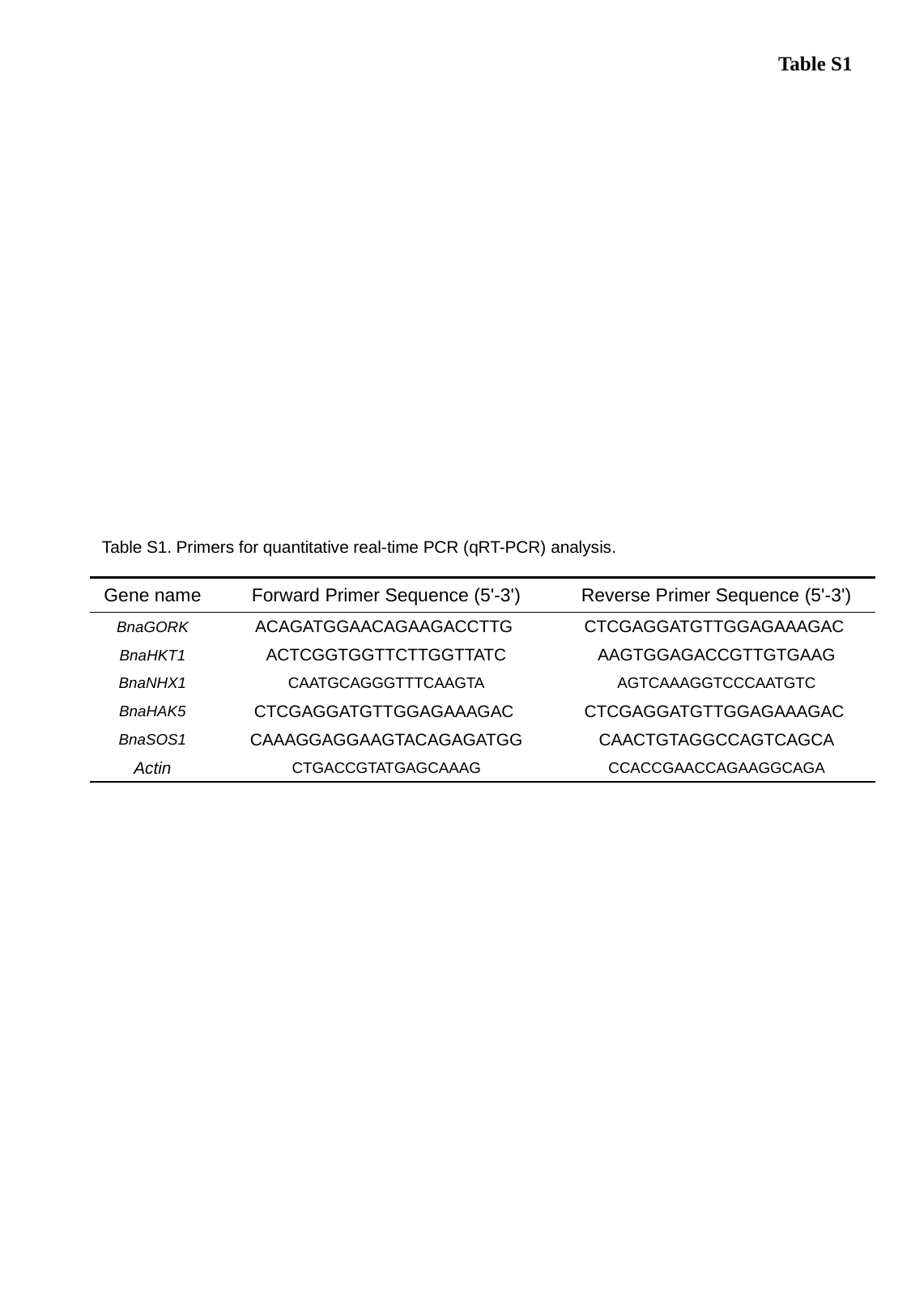

Table S1
Table S1. Primers for quantitative real-time PCR (qRT-PCR) analysis.
| Gene name | Forward Primer Sequence (5'-3') | Reverse Primer Sequence (5'-3') |
| --- | --- | --- |
| BnaGORK | ACAGATGGAACAGAAGACCTTG | CTCGAGGATGTTGGAGAAAGAC |
| BnaHKT1 | ACTCGGTGGTTCTTGGTTATC | AAGTGGAGACCGTTGTGAAG |
| BnaNHX1 | CAATGCAGGGTTTCAAGTA | AGTCAAAGGTCCCAATGTC |
| BnaHAK5 | CTCGAGGATGTTGGAGAAAGAC | CTCGAGGATGTTGGAGAAAGAC |
| BnaSOS1 | CAAAGGAGGAAGTACAGAGATGG | CAACTGTAGGCCAGTCAGCA |
| Actin | CTGACCGTATGAGCAAAG | CCACCGAACCAGAAGGCAGA |

## Slide 2
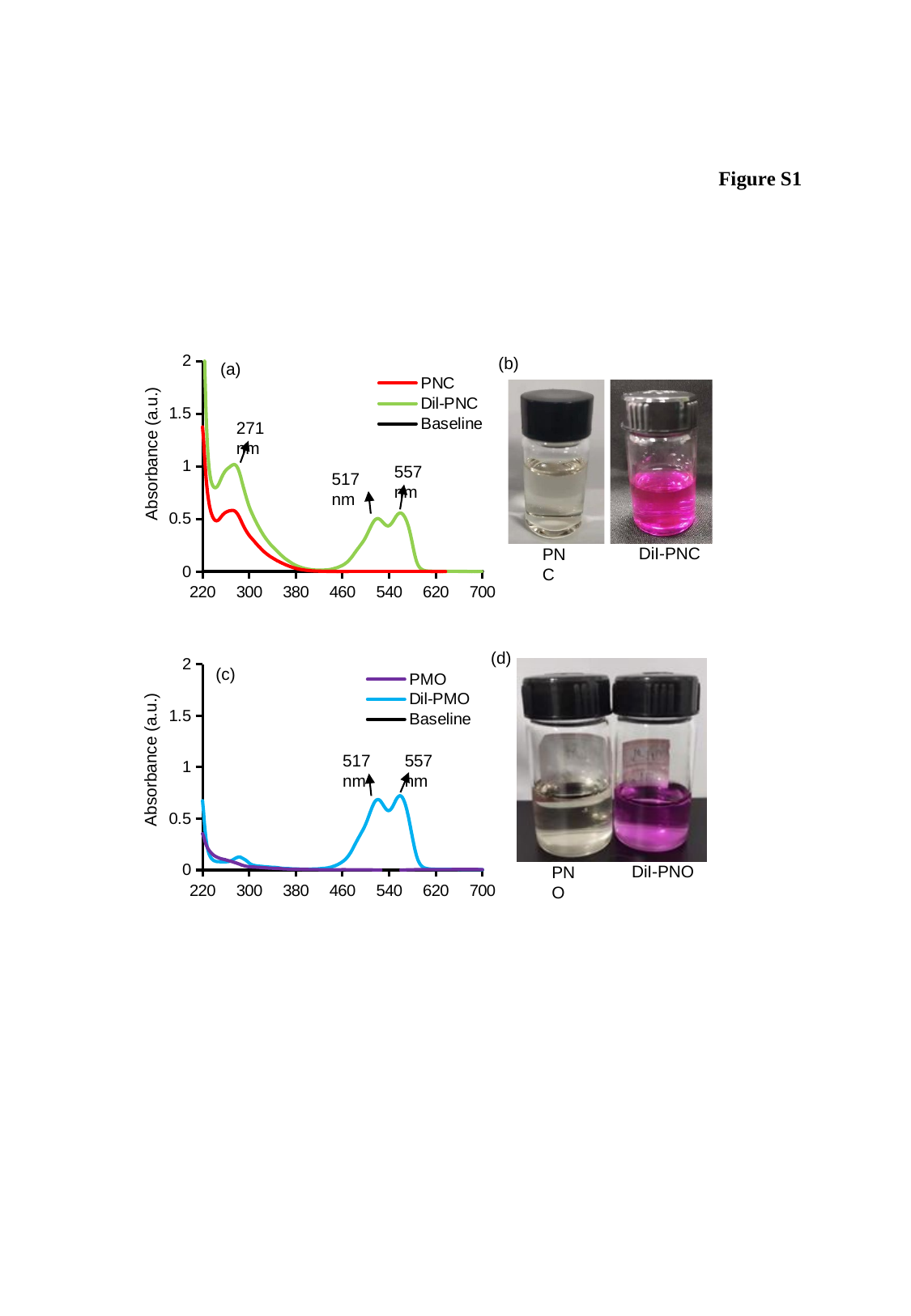

Figure S1
(b)
### Chart
| Category | PNC | Dil-PNC | Baseline |
|---|---|---|---|(a)
Absorbance (a.u.)
DiI-PNC
PNC
(d)
### Chart
| Category | PMO | Dil-PMO | Baseline |
|---|---|---|---|(c)
Absorbance (a.u.)
DiI-PNO
PNO
271 nm
557 nm
517 nm
517 nm
557 nm

## Slide 3
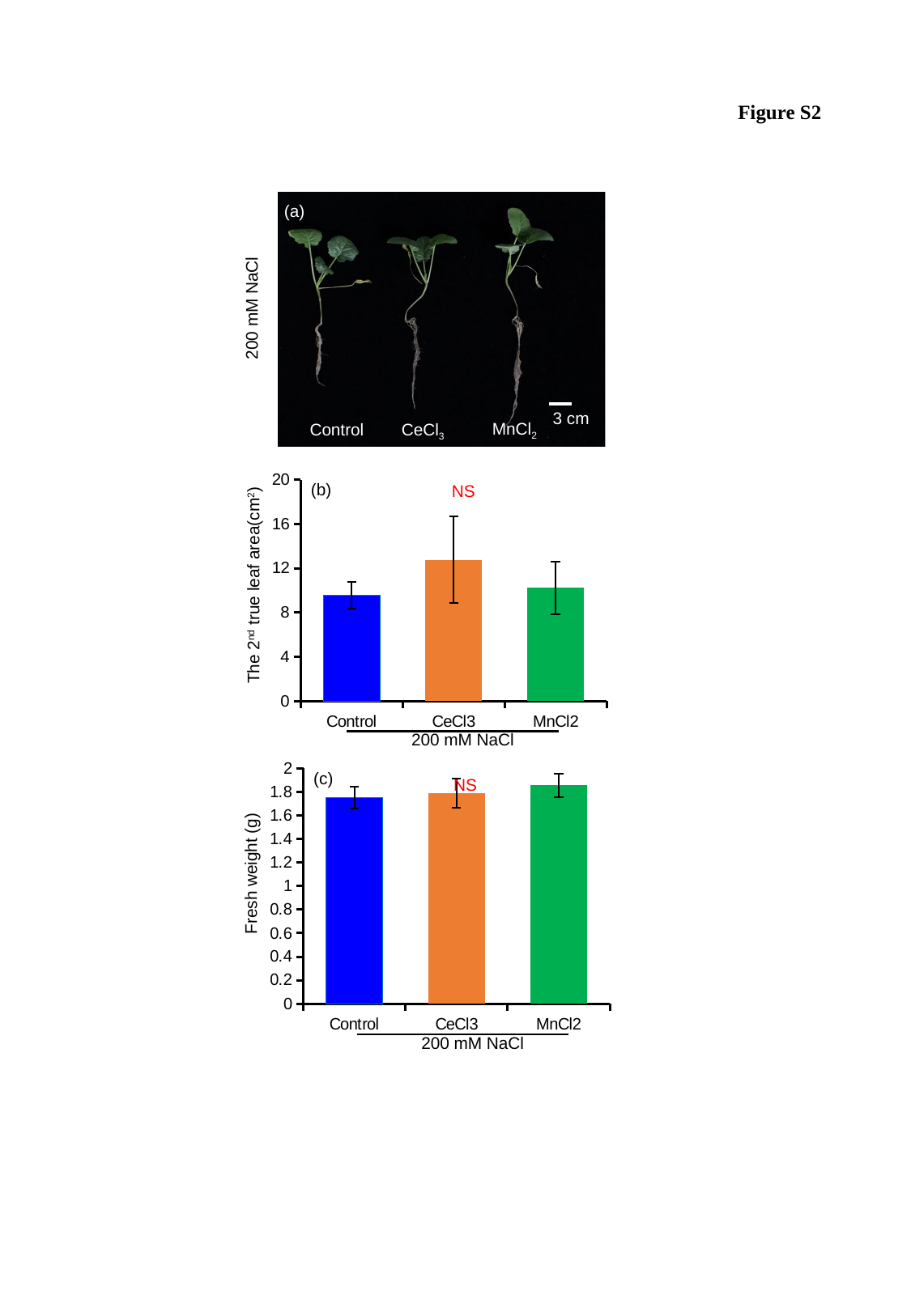

Figure S2
(a)
200 mM NaCl
Control
3 cm
MnCl2
CeCl3
### Chart
| Category | |
|---|---|
| Control | 9.561910498937156 |
| CeCl3 | 12.758888144236856 |
| MnCl2 | 10.230819215375497 |(b)
The 2nd true leaf area(cm2)
200 mM NaCl
### Chart
| Category | |
|---|---|
| Control | 1.75 |
| CeCl3 | 1.7914285714285716 |
| MnCl2 | 1.855714285714286 |(c)
Fresh weight (g)
200 mM NaCl
NS
NS

## Slide 4
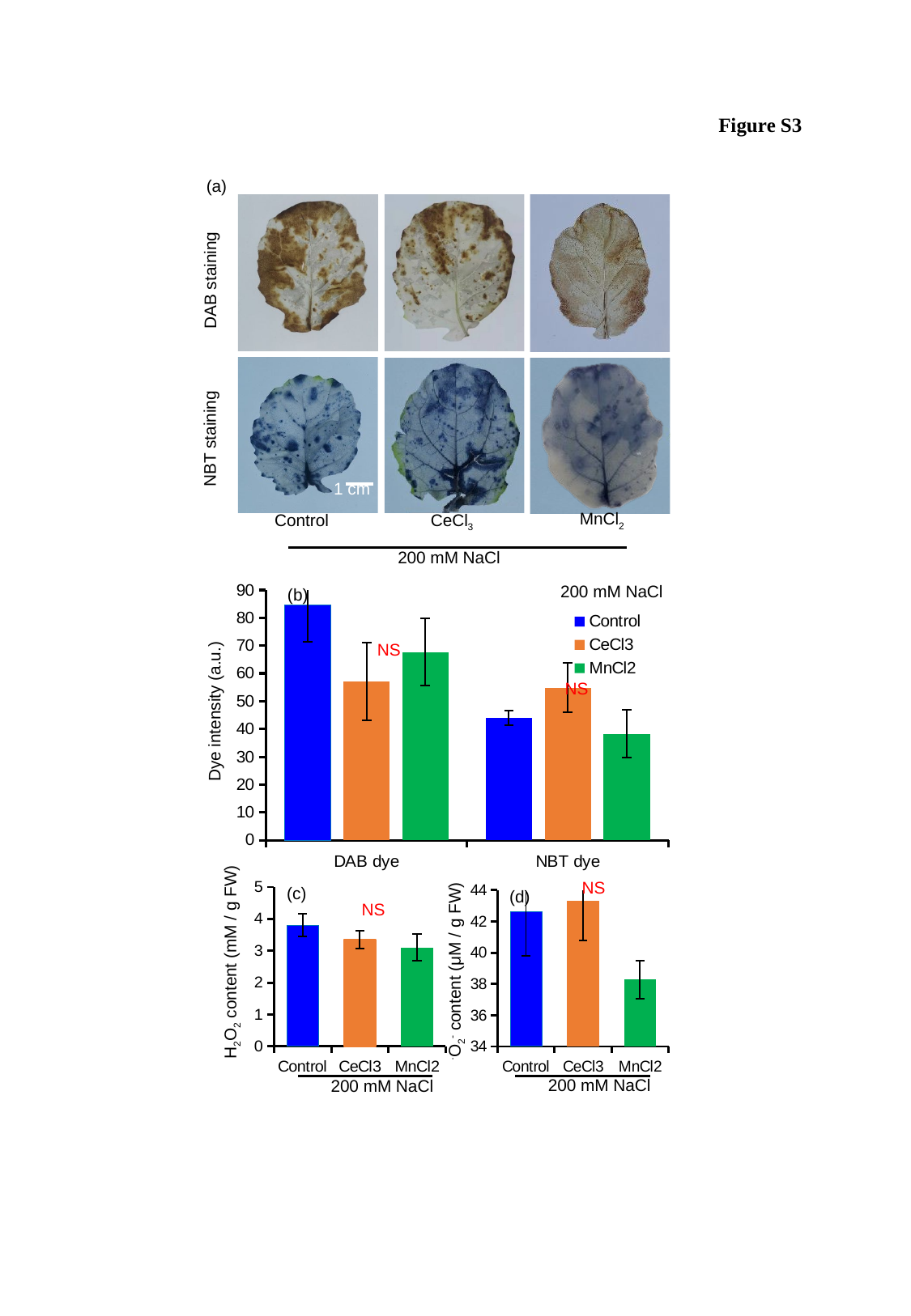

Figure S3
DAB staining
(a)
1 cm
NBT staining
Control
200 mM NaCl
MnCl2
CeCl3
200 mM NaCl
### Chart
| Category | Control | CeCl3 | MnCl2 |
|---|---|---|---|
| DAB dye | 84.66699620128756 | 57.11405783228518 | 67.633 |
| NBT dye | 43.890285344368756 | 54.869729196360595 | 38.310439933000005 |(b)
Dye intensity (a.u.)
H2O2 content (mM / g FW)
·O2- content (μM / g FW)
(c)
### Chart
| Category | |
|---|---|
| Control | 3.805269537425736 |
| CeCl3 | 3.3518458549222796 |
| MnCl2 | 3.100914149443561 |(d)
### Chart
| Category | |
|---|---|
| Control | 42.62068365478505 |
| CeCl3 | 43.327216704174546 |
| MnCl2 | 38.280516193235236 |200 mM NaCl
200 mM NaCl
NS
NS
NS
NS

## Slide 5
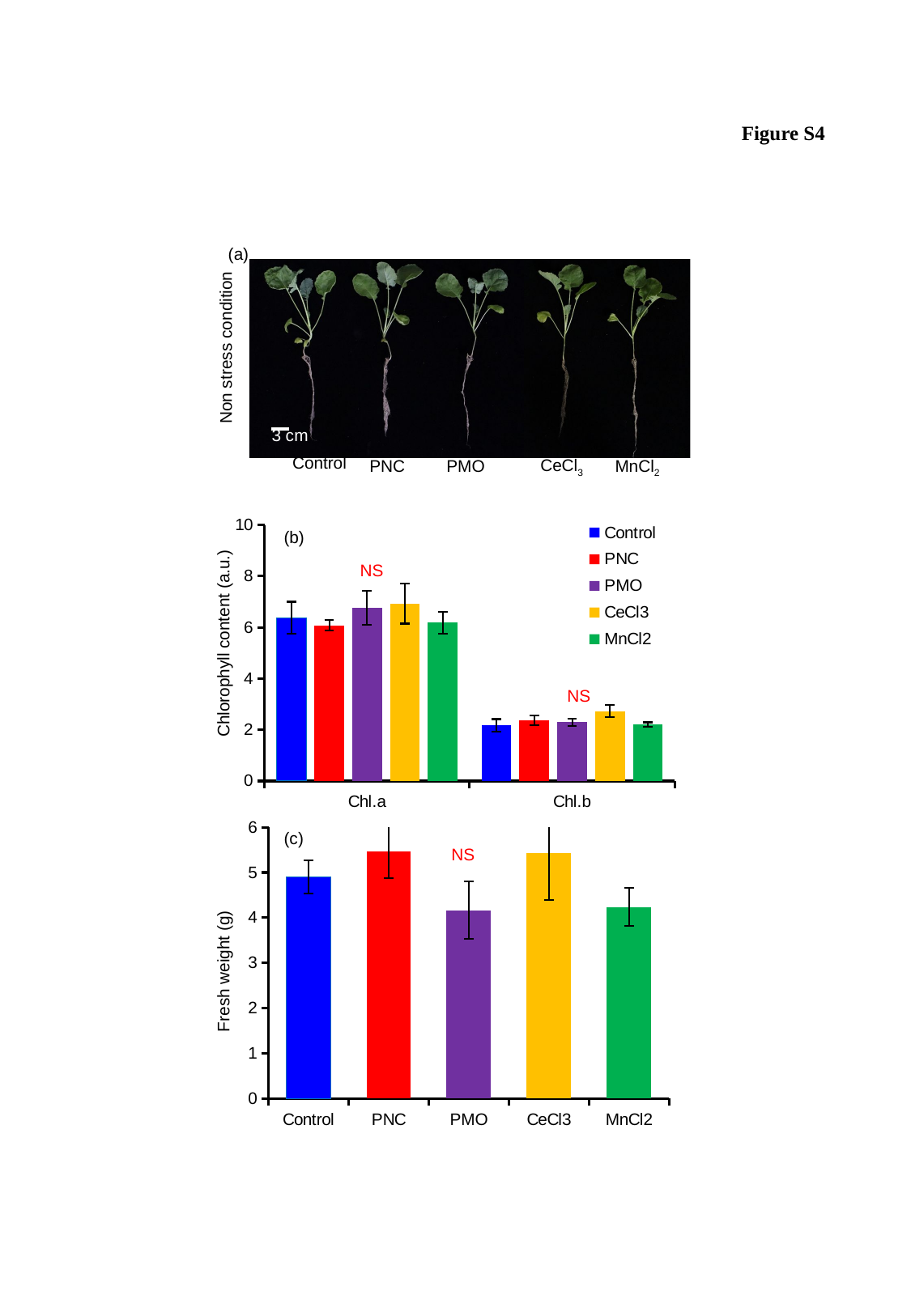

Figure S4
(a)
Non stress condition
3 cm
Control
CeCl3
PNC
PMO
MnCl2
### Chart
| Category | Control | PNC | PMO | CeCl3 | MnCl2 |
|---|---|---|---|---|---|
| Chl.a | 6.3795605 | 6.073758799250108 | 6.759854333333333 | 6.922136428154564 | 6.190760666666667 |
| Chl.b | 2.1713734999999996 | 2.37264756825861 | 2.2971562499999996 | 2.72210245806367 | 2.1954349999999994 |Chlorophyll content (a.u.)
(b)
### Chart
| Category | |
|---|---|
| Control | 4.898333333333333 |
| PNC | 5.463333333333334 |
| PMO | 4.163333333333333 |
| CeCl3 | 5.43 |
| MnCl2 | 4.234999999999999 |(c)
Fresh weight (g)
NS
NS
NS
